# Linking Auditory Brainstem Neural Stability to Parent-Reported Autistic Traits in School-Age Children

**DOI:** 10.3390/brainsci16050535

**Published:** 2026-05-19

**Authors:** Devon Pacheco Major, Emily Cary, Erin Matsuba, Natalie Russo, Beth Prieve

**Affiliations:** 1 Department of Communication Sciences and Disorders, Syracuse University, 621 Skytop Rd. Suite 1200, Syracuse, NY 13244, USA; baprieve@syr.edu; 2 Department of Psychology, Syracuse University, 430 Huntington Hall, Syracuse, NY 13244, USA; emily.cary@osumc.edu (E.C.); erin.matsuba@childrens.harvard.edu (E.M.); nrusso@syr.edu (N.R.)

**Keywords:** neural stability, autism, electrophysiology, auditory brainstem response

## Abstract

**Highlights:**

**What are the main findings?**

- Parents’ endorsement of autistic traits is related to the neural stability of auditory brainstem responses in school-aged children.
- This association exists regardless of whether the auditory brainstem response is evoked by speech or non-speech stimuli.

**What are the implications of the main findings?**

- Unstable auditory processing is associated with greater parent-reported autistic traits.
- These findings suggest that neural stability of speech and non-speech evoked auditory brainstem responses may reflect individual differences related to autistic traits, although further research is needed to determine the clinical and mechanistic significance of this relationship.

**Abstract:**

**Background:** Neural stability, defined as trial-by-trial fluctuations in neural responses to the repetitive sensory input, is an indicator of neural processing stability. The auditory brainstem response (ABR) can provide an electrophysiological measure of neural stability. Findings on neural stability differences between autistic and neurotypical individuals are inconsistent, potentially due to methodological differences and sample heterogeneity. This study aimed to investigate the relationship between neural stability in the brainstem and autistic traits in a group of children with and without a diagnosis of autism. We examined whether the degree of neural stability differs based on the evoking stimulus and response component analyzed, and whether neural stability relates to parent-reported autistic traits, as measured by the Autism Spectrum Quotient (AQ) and social responsiveness scale-2 (SRS-2). **Methods:** In total, 41 participants had usable click ABRs and 34 had usable sABRs. Neural stability was quantified using Pearson correlation analyses between binaurally evoked subaverage ABR waveforms. Parent-reported measures of autistic traits were collected. **Results:** Neural stability differed across ABR components, with the click ABR being significantly more stable than sABR components. Decreased neural stability is significantly related to autistic traits measured by the AQ but not the SRS-2. There was no significant response component by AQ interaction. **Conclusions:** Neural stability in the auditory brainstem pathway is linked to individual differences in autistic traits measured by the AQ but not the SRS, implying that early sensory processing neural stability may be related to broader features of autistic traits rather than social communication alone.

## 1. Introduction

Neural stability, defined as trial-by-trial fluctuations in neural responses to the same sensory input, is an indicator of efficient processing of external stimuli. Decreased neural stability is interpreted as increased noise within neural systems resulting from diminished synchronization across neural populations [1,2]. This reduced stability has been linked to atypical behavioral outcomes, such as decreased attention [3] and cognition [4], and has been observed across developmental and neurocognitive conditions, including autism [5,6,7].

Autism spectrum disorder is a highly heterogenous, neurodevelopmental condition characterized by varying degrees of impairments in social communication, social reciprocity, and the presence of restricted interests and repetitive behaviors [8,9]. This heterogeneity poses challenges for traditional group comparison designs, which can obscure meaningful individual differences. Additionally, autistic traits are continuously distributed across the general population [10,11,12]. Therefore, an analysis that examines autism-related characteristics across autistic (the preferred terms of most people diagnosed with autism are ‘autistic person’ and ‘person on the autism spectrum’; with respect to these preferences, these terms will be used to refer to individuals with a diagnosis of autism spectrum disorder) and non-autistic individuals can reveal brain–behavior relationships that may otherwise be obscured in categorical group comparisons [13].

**Figure 1:**
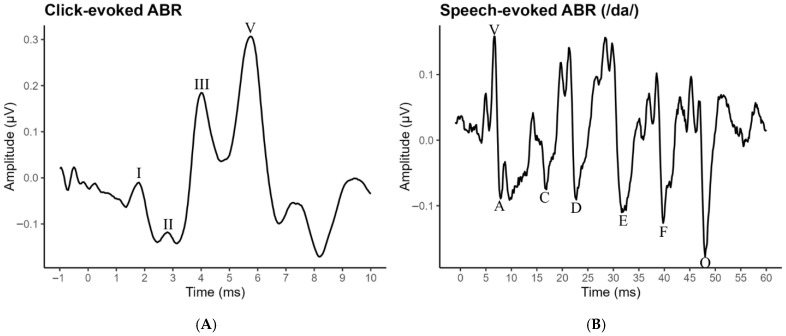
Click- and speech-evoked auditory brainstem responses. Note: This figure displays click- and 40 ms /da/-evoked ABRs in the time domain with hallmark peaks labeled in panels (**A**,**B**), respectively. The click ABR consists of wave I, III, and V and the sABR consists of V, A, C, D, E, F, and O.

Within the subcortical auditory brainstem pathway, the auditory brainstem response (ABR) can be used as a physiological indicator of neural stability. The ABR is an electrophysiological potential that captures synchronized neural activity in response to brief auditory stimuli. The ABR is commonly evoked by clicks (click ABR) or consonant–vowel clusters as speech ABR (e.g., /da/; sABR). The click ABR, depicted in Figure 1A, consists of five main waveforms following stimulus onset, primarily reflecting neural synchrony to the onset of sound in the auditory nerve (waves I and II), the cochlear nucleus and superior olivary complex (wave III), the lateral lemniscus (wave IV; not shown in Figure 1A), and the lateral lemniscus and inferior colliculus (wave V; for review see [14]).

An ABR evoked by a speech token can provide insight into the brainstem’s ability to encode speech-like features. The sABR evoked by a 40 ms synthetic /da/ syllable comprises seven waves (V, A, C, D, E, F, and O). Figure 1B depicts an sABR waveform with the waves labeled and the various response components partitioned, which include the complete response (0–55 ms), the onset (5–10 ms), the sustained portion (22–40 ms), and the offset (45–50 ms). Waves V and A, primarily generated by the lateral lemniscus and inferior colliculus, reflect the neural response to sound onset resulting from the initial friction in the production of the stop consonant /d/. Wave C, though not always present, signifies the transition from consonant to vowel or voicing onset [15]. Wave O marks the transition from stimulus offset to the absence of sound. These waves represent the transient segments of the stimuli, with their timing influenced by the acoustic filter characteristics, which include the speech articulators in natural speech [16]. Waves D, E, and F, correspond to the sustained portion of the response and are associated with the acoustic source of the stimuli: vocal fold vibration. This sustained part of the response, known as the frequency following response (FFR), reflects the neural phase locking to the fundamental frequency (*f*_o_) and its harmonics in the eliciting stimuli [15,17]. Temporally, the intervals between these peaks correspond to the wavelength of *f*_o_ [16]. Although there is some debate regarding the neural generators of the FFR, evidence suggests it arises mainly from the inferior colliculus [18]; but see [19].

Traditionally, ABR analyses focus on waveform latencies, where even small deviations from normative timing are considered indicative of meaningful differences in behavioral functioning [20]. Because the stability of the ABR waveform across repeated presentations reflects the precision of neural phase locking, the ABR can also be used to quantify neural stability (also referred to as response consistency) by calculating the linear relationship (Pearson correlation) between equal blocks of recorded ABR waveforms to repeated stimuli [20,21,22,23]. This results in an r value from 0 to 1, reflecting the consistency of the neural responses, with r values closer to 0 indicating less stable responses. Although ABRs are too noisy to be assessed at the trial level, support that this linear relationship reflects trial-by-trial neural stability comes from studies showing that the calculated stability metric is consistent when obtained using random samples of trials in a recording, interleaving the trials, or using separate averages for the calculation [24,25,26].

Recent work on ABR stability has increasingly pointed to the importance of stable subcortical processing of speech sounds for language development [24,27,28,29,30,31]. Collectively, this body of research suggests that when neural responses to sound are stable and repeatable, they support the formation for robust auditory representations that are critical for high-level language abilities. Referred to as the *Auditory Stability Hypothesis*, this work suggests that decreased stability in neural encoding across repeated instances of the same speech sound disrupts the establishment of consistent auditory representations [24]. In turn, this neural instability can compromise higher-level processes that rely on precise auditory input, including language perception and learning. In school-aged children, decreased neural stability in the auditory brainstem has been associated with dyslexia [24], reduced phonetic discrimination and syntactic performance [30], and increased pragmatic language violations [21]. For example, Tecoulesco et al. (2020) demonstrated a significant association between unstable FFRs and poor phonetic discrimination and syntactic performance [30]. Patel et al. (2022) found that decreased neural stability correlated with greater pragmatic language violations (*r* = −0.53, *p* < 0.001), although it is essential to note the relationship tested did not survive a Bonferroni correction for multiple comparisons [21]. Together, these findings suggest that neural stability in the auditory brainstem may relate to broad language and communication difficulties rather than a specific language mechanism.

While language and social skills, such as eye contact, reflect important characteristics of the autistic phenotype, autism is a highly heterogeneous condition that varies as a function of age, cognitive ability, autistic features, and the level of support needs. Autistic traits encompass far more than language abilities, spanning sensory reactivity, attention, and imagination. For example, differences in attention, a trait associated with autism, may be relevant to neural stability as reduced attention has been linked to decreased stability [3]. The extent to which subcortical neural stability is related to autistic traits, beyond aspects of language, is unknown. It is plausible that reduced stability in early auditory signal processing, limits the formation of robust auditory representations, which in turn has cascading effects that contribute to behavioral differences extending beyond language. Additionally, the evidence concerning differences in the degree of neural stability in autistic individuals compared to non-autistic individuals is mixed. Some studies report significantly decreased stability in both click ABRs and sABRs in groups of autistic individuals compared to non-autistic individuals [21,32], while others find no differences between groups [30]. These mixed findings may not only reflect methodological factors, such as the stimuli used in evoking ABRs and the number of averages collected, but also variability in participant characteristics including IQ, developmental differences, attention, sensory sensitivities, and heterogeneity of autistic traits. Such factors may be associated with neural stability independent of diagnostic status and are not uniform across studies. It is possible that reduced stability is associated with a specific dimension of autistic traits (beyond language) rather than diagnosis per se, and that heterogeneity within samples may determine whether significant relationships are observed. Taken together, this variability can obscure underlying effects, reduce statistical power, and contribute to inconsistent results across studies, particularly when samples are not sufficiently large or well characterized to account for these sources of variance. Therefore, it is important to examine whether neural stability is associated with autistic traits, beyond language abilities and across diagnostic boundaries. This approach is novel in that it shifts the focus from group-level diagnostic comparisons to dimensional variation in traits. Addressing this question could clarify whether auditory stability relates to broader autistic features rather than language-specific deficits, offering new insight into sensory processing differences associated with autistic traits in a dimensional manner.

There is no electrophysiological measure of autism; diagnosis is primarily based on parent/caregiver reports and behavioral assessments. Relevant to the current study are the Autism Spectrum Quotient (AQ) and the social responsiveness scale-2 (SRS-2). The AQ is a 50-item survey and is widely used in both clinical and research settings to assess traits associated with autism, specifically those related to social cognition, attention, and imagination [33]. AQ scores are normally distributed within the general population [11,12,33]. The SRS-2 is a 65-item rating scale that measures deficits in social behavior, including social awareness, social cognition, social communication, and social motivation, as well as restricted and repetitive behaviors (for review, see Bruni, 2014 [34]). Importantly, these scores also exhibit a normal distribution throughout the general population [10]. Although the AQ and SRS-2 surveys are similar, there are important differences. The AQ primarily captures autistic and cognitive tendencies, including attention to detail, attention switching, and imagination, which are not directly assessed by the SRS-2. In contrast, the SRS-2 focuses on the behavioral manifestation of autistic traits in everyday social contexts, including social motivation and restricted and repetitive behaviors, domains that are not explicitly represented in the AQ. Importantly, the SRS-2 places greater emphasis on social communication behaviors, such as conversational reciprocity, pragmatic language use, and responsiveness to social cues. These all rely on the functional use of language in social interaction. As a result, while the SRS-2 is not a direct measure of language ability, it is likely more closely related to language-mediated behaviors than the AQ and therefore, based on previous work, may be more likely to be related to neural stability.

In addition to exploring the relationship between neural stability and autistic traits, it is important to clarify whether the degree of stability differs across the temporal components of click- and /da/-evoked ABRs. Prior research has focused primarily on the stability of the /da/-evoked frequency following response (FFR) [21,30]. To our knowledge, no work has investigated whether the stability differs between the onset, sustained, and offset components of the ABR, nor examined whether the stability of these components is differentially related to autistic traits. The latency components of the sABR reflect the processing of different sound properties. The offset response reflects encoding of sound termination and contributes to temporal abilities, such as duration discrimination and perceptual grouping [35]. Thus, stability across these components may reflect distinct underlying neural mechanisms, each of which may differentially relate to sensory and perceptual characteristics associated with autistic traits.

While many group comparison designs might be negatively impacted by the inherent heterogeneity of autistic samples, here we take a different approach where we capitalize on that heterogeneity. We assess the relationship between ABR stability and features of autism that are present in autistic individuals and, to a lesser degree, in neurotypical children, as measured by the SRS-2 and AQ. In doing so, this study addresses two primary aims. First, it investigates whether the degree of neural stability, operationally defined as the degree of auditory brainstem response consistency, differs significantly depending on the type of evoking stimulus and the latency of response measurement. Second, it examines whether neural stability is related to autistic traits, as measured by the AQ and the SRS-2. The central research question is the following: to what extent is subcortical neural stability, measured via ABR, related to parents’ endorsement of autistic traits in a combined sample of school-aged children with and without autism, and does this relationship vary by ABR-evoking stimuli or components? We hypothesize that autistic traits, indexed by both the AQ and the SRS-2, will be related to neural stability across the full sample of autistic and non-autistic school-aged children, and that these relationships will differ as a function of the response component. Based on prior work linking language-related abilities to reduced neural stability [21,24,30], we predict that greater endorsement of autistic traits, as measured by the SRS-2, which captures how autistic traits manifest behaviorally in everyday social interaction and social communication, will be associated with reduced neural stability. We further predict that autistic traits measured by the AQ, which indexes broader characteristics and individual preferences of social and cognitive styles, will also be related to neural stability, consistent with the idea that early stability of auditory processing supports the formation of robust auditory representations with consequences for behavior. Given that the FFR component of the sABR exhibits poorer neural stability in autistic individuals compared to neurotypical individuals, we expect that the stability of the sustained portion of the response will be related to autistic traits. Additionally, because both the click ABR and the V/A complex of the sABR are reflective of an onset response, we predict that if one is associated with AQ or SRS scores, the other will be as well. These findings will enhance our understanding of neural instability and its behavioral correlates across both autistic and neurotypical individuals, suggesting that even in individuals without an autism diagnosis, neural stability is linked to autistic traits more broadly, rather than language specifically. Finally, identifying which response component’s stability is most associated with autistic traits can guide future research on neural stability and its impact on auditory processing in relation to autism.

## 2. Methods

This study was approved by the Institutional Review Board (IRB) at Syracuse University. Participants were recruited through the Center for Autism Research and Electrophysiology (CARE) Laboratory. Caregivers provided written informed consent, and the children provided written informed assent prior to participating. Participants completed the full study in multiple sessions. Participants completed the hearing assessment and ABR tasks at the Pediatric Audiology Laboratory in one session. In another two–three sessions, participants completed an IQ assessment and EEG tasks at the CARE Laboratory. Parents completed questionnaires as their child participated in the sessions. Participants were compensated for their time. The sABR data have been analyzed in a different manner in a subset of participants and published by Matsuba et al. (2022) [36]. The EEG findings and methodology is not reported here but has been reported by Matsuba et al. (2022) [36] and Cary et al. (2023) [37].

A total of 44 school-aged children (6–16.9 years), consisting of 18 autistic children and 26 neurotypical peers, were enrolled. Table 1 displays participant demographic information, including race, education level, gender, and highest completed parental education. As discussed in Section 3, not all participants were able to complete all aspects of the study; therefore, the number of participants who completed each dataset is reported. Autism diagnoses were confirmed by a research-reliable licensed psychologist at the CARE laboratory using the Autism Diagnostic Observation Schedule, Second Edition (ADOS-2), the Autism Diagnostic Interview-Revised (ADI-R), and clinical judgment based on DSM-5 criteria. Participants were excluded from the study if they had a reported medical history positive for epilepsy; neurological, genetic, psychiatric, or learning disorders; or hearing loss, defined by a behavioral threshold greater than or equal to 25 dB HL at two or more octave frequencies between 250 and 8000 Hz, or an elevated threshold (≥25 dB HL) at one frequency and an abnormal tympanogram (peak-compensated static admittance magnitude <0.2 or >1.4 mmho and middle-ear pressure <−150 or >+25 daPa). For inclusion in the study, participants had to be between the ages of 6 and 17 years old, verbal English speakers, and have a full-scale IQ above 80.

### 2.1. IQ Assessment

IQ assessment was performed using the Wechsler Abbreviated Scale of Intelligence, Second Edition (WASI-II). The WASI-II is an abbreviated measure of verbal, non-verbal, and general cognitive intelligence for individuals aged 6 to 90 years [38]. The WASI-II yields the following composite scores: full-scale IQ (FSIQ), verbal comprehension index (VCI), and Perceptual Reasoning Index (PRI). The PRI assesses visuospatial skills and is less reliant on verbal communication. The VCI measures a participant’s verbal conception formation, verbal world knowledge, crystallized intelligence, and degree of language development. All scores are standardized (M = 100, SD = 15).

### 2.2. Parent Questionnaires

While the children were engaged in the experimental tasks, the parents were asked to complete a series of questionnaires; those relevant to the current study include the AQ and SRS-2. For the AQ, parents completed one of two forms depending on the participant’s age: the parent-report Child AQ for ages 4–11 [39] or the Adolescent AQ for ages 12–15 [40]. Participants aged 16 and older were asked to complete the self-report Adult AQ before or after the experimental task [33]. Each form addresses the same content, but the items on each form are adapted for different developmental levels. To be consistent with the other versions of the AQ, scores on the Child AQ were converted, such that item scores of 0 or 1 were converted to 0 and scores of 2 or 3 were converted to 1, to get a total AQ score ranging from 0 to 50. Higher scores indicate a greater degree of autistic traits. A score of 32 or higher is highly predictive of autism. The AQ has good convergent validity [41], high internal validity with a Cronbach’s alpha of 0.74, and strong test–retest reliability of 0.82 (Pearson’s correlation coefficient) [42]. Consistent with prior work, AQ scores from all three forms were pooled for the primary analyses [36,37]. To confirm that including self-report data from older participants did not influence the results, a supplemental analysis excluding Adult AQ responses was conducted; these results are presented in the Supplementary Material.

For the SRS-2, parents were asked to rate various statements on a 4-point Likert-type scale, ranging from “not true = 1” to “almost always true = 4”. Scores from the SRS-2 were reported as T-scores (M = 50, SD = 10). A score of 76 or higher is considered severe, indicating clinically significant deficits in social functioning. Scores ranging from 66 to 75 are categorized as moderate, 60–65 indicate mild to moderate deficiencies, and scores below 59 are not indicative of a potential autism diagnosis [34]. For the SRS-2, parents in this study were asked to complete the school-aged form of the SRS-2 used for participants aged 4–18 years. The overall total score is the most reliable measure for social deficits related to autism [34] and was used as the measure from the survey reported here. The SRS, from which the SRS-2 is derived, has good psychometric properties. The test–retest reliability of the SRS ranges from 0.72 to 0.97 (intraclass correlation) and the internal consistency of the measure ranges from 0.91 to 0.97 (Cronbach’s alpha). The specificity and sensitivity of the SRS total score of 85, when differentiating between autistic and neurotypical individuals, is 0.81 and 0.73, respectively. The SRS has moderate to good convergent validity [43]. Although the SRS-2 and AQ are different, there is good convergent validity between the two surveys supported by the significant correlation between ratings of the SRS and AQ (r = 0.64, *p* = 0.00) [44].

### 2.3. Hearing Assessment

Prior to electrophysiological recordings, all participants underwent a routine hearing assessment, which consisted of air-conduction behavioral thresholds at octave frequencies ranging from 250 to 8000 Hz (including inter-octaves 3000 and 6000 Hz). Conditioned play audiometry was employed to elicit threshold responses if a participant was unable to complete standard behavioral audiometric testing. If behavioral thresholds were elevated (≥25 dB HL) at any frequency tested, tympanometry was performed on the participant to assess their middle ear’s status.

### 2.4. Electrophysiology

The Intelligent Hearing System, SmartEP, was used for stimulus presentation and recording. The click ABR was evoked using a 100µs broadband click presented at 70 dB nHL (98 dB pSPL) at a rate of 27.7/s using condensation polarity. The sABR was evoked by a 40 ms synthetic /da/ with alternating polarity at a rate of 11.1/s. The /da/ stimuli were presented at 63 dB nHL (80 dB SPL). All stimuli were presented binaurally with insert ER-3A earphones separately placed in the right and left ears. Stimuli were calibrated in an HA-1 coupler coupled to a sound level meter following standard procedures for calibrating insert earphones. A two-channel montage, right and left channels, was used to record a click ABR and an sABR from four scalp electrodes: right and left mastoids (inverting), forehead (non-inverting), and low forehead (ground). The four areas were prepped with an alcohol pad and Nuprep gel. Tab electrodes were placed on the washed areas. Electrode impedances were < 3 kohms and within 1 kohm of each other.

Responses recorded over the right channel were the ipsilateral recordings from right ear stimulation; similarly, the responses recorded over the left channel were the ipsilateral recordings from the left ear stimulation. Both ears were stimulated synchronously during binaural stimulation, and right and left ipsilateral responses were recorded simultaneously. Responses were amplified with a gain of 100,000 and bandpass filtered from 100 to 3000 Hz (responses were recorded on two different computers utilizing the same equipment and set-up, except for online vs offline filtering of the sABR. Nine of the usable sABR data were bandpass filtered offline and 28 were bandpass filtered online. An independent *t*-test (unequal variance) indicated that there was no difference in online vs offline filtering on the degree of neural variability (*p* = 0.608). Trials exceeding ±35 μV were rejected from the running average and were not included in the final analysis. Recording was complete when at least two averages containing 1500 low-noise runs were collected over a 60 ms and a 25 ms window to speech and click stimuli, respectively, for each channel.

The presence of the click ABR was established via visual inspection of the presence of waves I, III, and V, with consensus reached 100% of the time by two clinically trained doctoral students who evaluated the waveforms independently. The presence of the sABR was assessed by analyzing the spectral content of the FFR portion in MATLAB to determine whether the response amplitude exceeded the noise floor (i.e., was present), consistent with the previous literature (for review, see Picton et al., 2003 [45]). When click ABR waveforms were not identifiable or the amplitude of the sABRs were not above the noise floor, the data were excluded from the final analysis.

The root mean square (RMS) of the interval prior to stimulus presentation was calculated to control for individual differences in prestimulus noise. For the click ABR, the prestimulus interval was 0.8 ms and for the sABR, it was 5 ms. The length of the prestimulus interval was calculated by taking 10% of the length over which the response was analyzed (8 ms and 55 ms for click and speech ABR, respectively; Hall, 2007 [14]). The prestimulus noise was correlated with all variables to determine if it should be entered into the statistical models as a covariant, as has been previously reported [46]. Auditory brainstem response data, which was stored in two buffers during online recording, and then downloaded and analyzed offline to calculate the degree of neural stability.

### 2.5. Calculation of Neural Stability

Neural stability was quantified using the Pearson product–moment correlation, a standard measure of ABR consistency [20,24,29]. This metric reflects the degree to which two independently averaged responses to the same stimulus exhibit similar morphology: higher correlations indicate more repeatable (i.e., stable) neural activity, whereas lower correlations indicate reduced stability.

Because speech stimuli were presented in alternating polarity and responses were recorded simultaneously from right and left channels, it was necessary to structure the subaverages such that the resulting correlation reflected true neural stability without being confounded by polarity-specific timing shifts, channel differences, or recording-order. As detailed in Supplementary Material S1 each channel (channel A and B) produced two polarity-specific buffers of 1500 sweeps (condensation and rarefaction). To eliminate polarity-dependent differences in waveform morphology, we constructed two average responses by pairing condensation trials from one channel with rarefaction trials from the opposite channel and vice versa. This procedure yielded two subaverages (n = 3000) that contained equal numbers of condensation (n = 1500) and rarefaction sweeps (n = 1500), balanced responses recorded in right (channel A) and left (channel B) channels, as well as the first and second collection, and were derived from non-overlapping sets of trials. Thus, any remaining differences between the two averaged waveforms are attributed to reduced neural stability rather than stimulus polarity, recording order, or channel effects. For click ABRs, which were collected using a single polarity, the same cross-channel averaging procedure was applied to maintain methodological consistency and eliminate channel or recording-order variance. Pearson’s *r* was computed between the two resulting subaverage waveforms for each participant. Correlations were computed across specific time windows to isolate different response components: the click ABR (1–8 ms) and four sABR windows, the whole response (0–55 ms), onset (5–10 ms), FFR (22–40 ms), and offset (45–50 ms). Correlation coefficients were Fisher transformed (Zr) for statistical analyses.

### 2.6. Statistical Analysis

All statistical analyses were performed using IBM SPSS Statistics (version 17) and R v.4.4.2 [47]. Descriptive statistics, including the mean, median, range, and standard deviation, were computed in SPSS for binaural neural stability measures derived from the click ABR and the sABR, including the entire response, onset, FFR, and offset components. Descriptive statistics were also calculated for the prestimulus noise and for autistic trait measures.

Prior to addressing the primary research questions, preliminary analyses were conducted to ensure that neural stability did not differ as a function of ear of stimulation. To do this, a two-way repeated-measures analysis of variance (RMANOVA) was conducted in SPSS comparing right- and left-ear responses evoked by monaural stimulation. Additionally, relationships between prestimulus noise and neural stability measures (click ABR and each sABR component), as well as autistic traits (AQ and SRS), were assessed using Pearson correlation analyses. An independent samples t-test was also conducted to determine whether prestimulus noise differed between autistic and neurotypical participants.

Subsequent analyses examining the relationship between autistic traits and neural stability were conducted in R v.4.4.2 [47]. Data organization and visualization were performed using the tidyverse [48] and ggplot2 [49] packages. Linear mixed-effects models (LMMs) were fit using the lme4 package [50], with participants included as a random intercept to account for repeated measurements across ABR components. Statistical testing for fixed effects was conducted using lmerTest [51], which provides Satterthwaite-approximated degrees of freedom for *t*-tests. Model diagnostics were assessed using performance [52] and DHARMa [53], while estimated marginal means and post hoc contrasts were computed using emmeans [54]. To evaluate whether neural stability differed across ABR components and in relation to autistic traits, ABR components were included as a fixed effect and were sum-coded (effects coding), so that fixed-effect estimates reflected differences from the grand mean rather than in comparison to a single reference component. Autistic traits were examined using both the AQ and SRS and were entered into models as continuous predictors. Neural stability used the Fisher-transformed Z-scores. For each trait, a set of nested candidate models was fit using maximum likelihood estimation:

Model 0: Neural stability ~ component + (1 | participant).
Model 1: Neural stability ~ component + trait + (1 | participant).
Model 2: Neural stability ~ component × trait + (1 | participant).
Model 3: Neural stability ~ component × trait + age + verbal comprehension index (VCI) + (1 | participant).

Model selection was based on the Bayesian Information Criterion (BIC). When an ABR component was retained as a fixed effect, estimated marginal means and pairwise comparisons among components were conducted using Tukey-adjusted tests to control for multiple comparisons. Participants were included if they had at least one valid neural measure and a complete AQ or SRS score. Linear mixed-effects models were fit using maximum likelihood, which accommodates incomplete observations; therefore, listwise deletion across components was not applied, and sample sizes varied slightly across models.

## 3. Results

Although 44 participants enrolled in the study, a full dataset was not usable or collected from each participant. Two participants had atypical peripheral hearing in either one or both ears (n = 1 autistic, n = 1 neurotypical) and were therefore excluded. All other participants had behavioral thresholds equal to or less than 20 dB HL at octave frequencies from 250 to 8000 Hz. Electrophysiological data were incomplete for three participants, and binaural sABRs in five participants had excessive noise and did not pass the F-test. Therefore, 26 participants had a complete dataset, including electrophysiological data, a full audiometric test battery, all IQ and survey measures. In total, 41 participants had usable click ABRs and 34 participants had usable sABRs. There were no significant differences between participants with complete usable ABR measures and those with incomplete data in VCI (*p* = 0.42), AQ scores (*p* = 0.46), or SRS-2 scores (*p* = 0.09). Four participants (n = 2 autistic and n = 2 neurotypical) completed the self-report Adult AQ because they were older than 16 years of age. Table 2 displays descriptive statistics, including minimum, maximum, mean, standard error of the mean, and standard deviation, as well as the number of participants contributing to the data for each measure. VCI was moderately correlated with the SRS-2 (*p* = 0.009) and was not significantly correlated with the AQ (*p* = 0.064). Variance inflation factors indicated no evidence of problematic multicollinearity (all VIFs ≤ 1.24). There were no significant correlations between prestimulus noise and any neural response component or participant variable; therefore, prestimulus noise was not included in subsequent analyses. These correlation analyses are found in the Supplementary Material S2. In addition, prestimulus noise did not differ between autistic and neurotypical participants for either the sABR (*t*(32) = 0.00, *p* = 1.00) or the click ABR (*t*(39) = 0.07, *p* = 0.94).

### 3.1. Effects of Ear and Response Component

A two-way repeated-measures ANOVA was conducted to evaluate the effects of ear (left vs. right) and response component on neural stability. Mauchly’s test of sphericity indicated that the assumption of sphericity for response component was violated (χ^2^(9) = 83.67, *p* < 0.001); therefore, Greenhouse–Geisser corrections were applied (ε = 0.60). There was a significant main effect of the response component on neural stability (*F*(2.40, 76.64) = 11.71, *p* < 0.001), indicating that the degree of neural stability differed across ABR components. There was no significant main effect of the ear (*F*(1, 128) = 0.12, *p* = 0.73) and no significant ear-by-component interaction (*p* = 0.70). An additional RMANOVA including participant group (autistic vs. neurotypical) as a between-subject factor revealed no significant effects of the group (*F*(1, 1) = 0.32, *p* = 0.58) and no significant interactions involving the group. These findings support the use of binaural stimulation and the combination of right- and left-ear recordings for subsequent analyses of neural stability.

#### Linear Mixed-Effects Models of Neural Stability

To examine the relationship between neural stability and autistic traits while accounting for repeated measurements across ABR components, LMMs were fit with participants included as a random intercept. Neural stability (Fisher-transformed Z-scores) served as the dependent variable, ABR components were included as a fixed effect using sum coding, and autistic traits were examined separately using the AQ and SRS. Table 3 displays the BIC values for the AQ and SRS-2 models that were tested.

For the AQ, the total sample size was 40 participants. Model comparison based on BIC indicated that the model including ABR components and AQ as fixed effects fit the data best. Table 4 presents the complete model output. This model demonstrated a significant main effect of the AQ on neural stability (*β* = −0.023, *SE* = 0.008, *t* = −2.81, *p* = 0.008), indicating that higher AQ scores were associated with decreased stability (lower neural response consistency) across ABR components. Significant differences in neural stability were observed across ABR components. Tukey-adjusted comparisons indicated that click differed significantly from all other components, and that onset exhibited greater stability than both full sABR and offset. No other pairwise comparisons were significant. However, model fit did not improve with the inclusion of interactions between the AQ and response components, suggesting that the relationship between the AQ and stability did not significantly differ by component. Similarly, age and VCI did not account for additional variance beyond the AQ and components.

**Figure 2:**
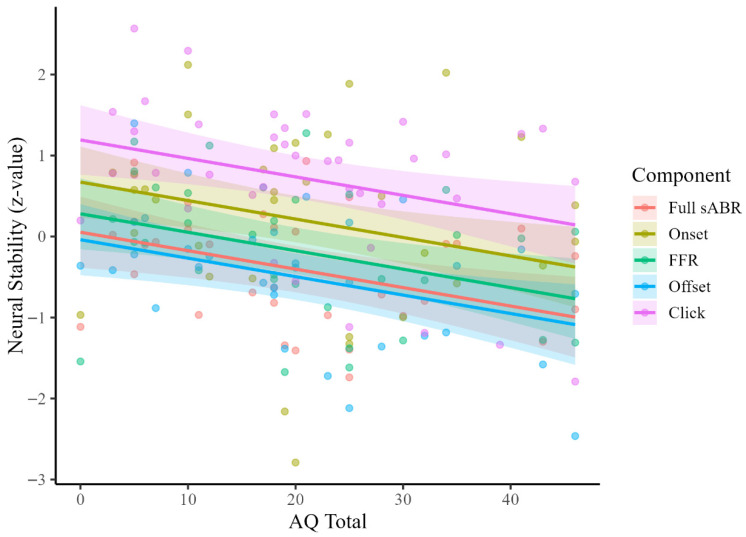
Linear mixed-effect model: neural stability and AQ. Note: This figure shows the relationship between AQ score and neural stability across response components, with individual data points, model-predicted regression lines, and 95% confidence intervals. Each response is color-coded according to the following: full sABR is red, onset is gold, FFR is green, offset is blue, and click ABR is purple. There is no significant interaction between response component and AQ total.

The marginal R^2^ for the best-fitting model indicated that fixed effects accounted for approximately 29% of the variance in neural stability, while the conditional R^2^ indicated that the full model, including random effects, accounted for approximately 58% of the variance. To quantify the unique contribution of the AQ, models were compared with and without the AQ as a predictor. The AQ accounted for an additional 7.7% of variance in neural stability beyond the response component effects (∆R^2^ = 0.077). Figure 2 depicts the relationship between the AQ score and neural stability across response components. Individual participant data points are shown alongside model-predicted regression lines and 95% confidence intervals for each component. Consistent with the LMM results, higher AQ scores were associated with reduced stability across all response components. As evidenced by the parallel slopes of the different components, there was no significant AQ-by-component interaction.

This analysis was repeated excluding the four participants who completed the self-report Adult AQ (n = 4). The analysis, which included only parent-report versions of the AQ, did not differ from the results reported above. The model demonstrated a significant main effect of parent-report only AQ on neural stability (β = −0.021, SE = 0.008, *t* = −2.51, *p* = 0.017), indicating that higher AQ scores were associated with decreased stability (lower neural response consistency) across ABR components. The full model results comparing the linear mixed models with and without the self-reported AQ data are included in the Supplementary Material S3.

For the SRS-2, the total sample size was 34. The best-fitting model, based on BIC, included ABR components as the only significant predictor. Models that included the SRS as a main effect or in interactions with components did not improve model fit relative to the component-only model. As with the AQ analyses, significant differences in neural stability were observed across ABR components, indicating component-specific stability differences independent of SRS scores.

An exploratory analyses was conducted to examine whether the association between the AQ and neural stability was due to a specific AQ subscore: *attention switching, attention to detail, communication, social skills, and imagination*. A model that included ABR component and the AQ score minus the subscore was compared to the full model which included the subscore. Likelihood ratio tests evaluated whether adding the subscale improved model fit. These tests indicated that none of the AQ subscales explained additional variance beyond the total AQ score (communication: χ^2^(1) = 1.24, *p* = 0.266; social skills: χ^2^(1) = 0.66, *p* = 0.416; attention switching: χ^2^(1) = 0.47, *p* = 0.491; attention to detail: χ^2^(1) = 0.03, *p* = 0.861; and imagination: χ^2^(1) = 0.01, *p* = 0.916).

## 4. Discussion

The present study analyzed ABR neural stability measured in a collapsed sample of 41 school-aged children with and without autism. The findings revealed significant differences in the degree of neural stability based on the response component analyzed and that reduced stability of the ABR, is associated with greater endorsement of parental traits on the AQ, regardless of which component is analyzed. However, there was no significant relationship found between parents’ endorsement of autistic traits measured by the SRS-2 and neural stability. These associations were not driven by participant age or VCI, as these variables did not improve model fit when entered into the models.

The stability of the click response differed significantly from that of all other response components, including the onset response. It was predicted that the onset component of the sABR and the click ABR would exhibit similar stability, as both are thought to originate from similar neural generators and reflect transient responses to rapid acoustic onsets [55]. Contrary to this prediction, however, the click response exhibited significantly greater stability than the onset response. Several factors may account for the overall differences in degree of neural stability between click ABR and sABR components. First, the stimuli differed in frequency composition. The click stimuli were broadband and may elicit a more robust and synchronous neural response than speech stimuli. Second, the duration of the analysis window differed across components; longer time windows, as used for sABR, may introduce greater variability, resulting in reduced stability. Finally, the presentation rate differed between the sABR and the click ABR. Stimulus presentation rate is known to influence ABRs, such as their morphology [23], and the difference in presentation rate between the click- and speech-evoked ABRs may have affected the observed degrees of stability between components. Importantly, however, although the degree of stability differed between components, the relationship between stability and the AQ did not differ as a function of the response component.

Previous research has often focused solely on analyzing the neural stability of the FFR component. The analysis of the FFR provides a measure of how well the neural system phase locks. However, this analysis is in terms of both timing and latency in reference to the evoking stimuli. Calculating neural stability is rooted in estimating the linear relationship between two subaverage response waveforms, providing insights into the trial-to-trial changes in neural fluctuations, irrespective of their relation to the *f*_o_. Therefore, in terms of neural stability, the other response components may provide similar insight into the stability of neural encoding as the FFR. Importantly, these findings indicate that the relationship between autistic traits and neural stability can be meaningfully assessed using click stimuli. Because click-evoked ABRs are routinely collected in clinical settings, this approach enhances the feasibility of translating neural stability metrics into clinically accessible assessment tools.

Given the results of the current study, evaluating neural response stability, regardless of component/stimulus (including click ABRs), reveals a similar relationship between parent-reported autistic traits, as measured by the AQ, and neural stability. Building on *the Auditory Stability Hypothesis [24]*, which emphasizes the importance of stable neural processing of speech sounds, the present findings extend this framework by suggesting that reduced stability in neural encoding across repeated presentations of both speech and non-speech sounds may hinder the formation of consistent auditory representations. Disruptions in these representations may, in turn, compromise processes that rely on precise auditory input, extending beyond language to encompass broader autistic traits.

Specifically, parents’ endorsement of autistic traits measured by the AQ, which include attention to detail, attention switching, and imagination, are associated with the stability of early auditory processing. In the current study, the SRS-2 and AQ scores were highly correlated (r = 0.88, *p* < 0.001). Therefore, it was expected that if one measure was related to neural stability, the other would be as well, but if there was a difference, it would be due to differences in what the surveys measured. These findings reveal that neural stability is related to parents’ endorsement of autistic traits measured by the AQ rather than the SRS-2. Therefore, neural stability was associated with parents’ endorsements of traits regarding attention switching, attention to detail, social skills, communication, and imagination. Because the AQ measures traits associated with attention while the SRS-2 does not, it is plausible that the association with neural stability is driven by parents’ endorsement of attention-related traits, although the exploratory analysis did not indicate the relationship was driven by the subscales *attention switching* or *attention to detail*. It was expected that the SRS-2 would be associated with neural stability based on prior findings linking neural stability to language; however, it is important to acknowledge that the SRS-2 is not a direct measure of language and therefore may not show the same relationship with neural stability as direct language measures do [30].

In adults, the AQ has better predictive ability than the SRS-2 [56]. Because this analysis was conducted across diagnostic boundaries rather than within a clinically defined autistic group, it is also possible that the AQ is more sensitive to dimensional variation in neural stability across the broader population. In contrast, it is possible that the SRS-2 may capture behavioral manifestations of autistic traits that become most strongly related to neural processing only after individuals meet diagnostic criteria for autism. Thus, neural stability across diagnostic boundaries may be more closely related to broader, trait-level characteristics measured by the AQ, whereas within clinically diagnosed groups, it may be more directly linked to the functional use of language in social interactions. Future studies with a greater number of autistic participants should investigate whether this holds true. Additionally, although both surveys assess parents’ endorsement of their child’s autistic traits, they differ in how items are framed. The AQ emphasizes a child’s preferences (e.g., preferring to do things with others rather than alone), whereas the SRS-2 focuses on observable behaviors (e.g., seems much more fidgety in social situations when alone). This distinction between preferences and observable characteristics may contribute to the differing associations between parents’ endorsement of autistic traits and neural stability. It is possible that a child’s behavior is shaped or modified over time through adaptation or social expectations. As a result, preference-based items (AQ) may be more directly associated with underlying neural stability, whereas behavior-based items that may have been influenced by compensatory strategies are not associated with underlying neural stability. It would be interesting to assess the association between neural stability and preference vs. observable traits in younger children who may not have developed compensatory strategies to test this theory. It is also important to acknowledge that, although the AQ was a significant predictor of neural stability, it accounted for only a modest proportion of unique variance (7.7%) beyond response component effects. This suggests that individual differences in autistic traits, as measured by the AQ, are meaningfully associated with neural stability, but that a substantial proportion of variance is driven by other factors not captured in the model.

Examining the relationship between neural stability and autistic traits across diagnostic boundaries was a strength of the current study. This analysis allowed for exploring heterogeneity not captured by conventional group comparisons. To that point, an exploratory, mixed repeated measures ANOVA was used to assess whether neural variability, when evoked binaurally, differed between autistic and neurotypical children. The analysis indicated that while neural variability varied by response component, the two groups did not exhibit significant differences or significant group-by-response component interaction, suggesting that the heterogeneity captured in the main analysis was not captured in a group comparison. The lack of between-group differences contrasts with some prior studies that reported less stable responses in autistic children when evoked via the right ear compared to neurotypical children [21,32]. The results of the exploratory analysis in the current study align more closely with Tecoulesco et al. (2020), who reported no difference in neural variability in the FFR portion of the sABR between autistic and neurotypical children [30]. The significant model found by combining autistic and neurotypical participants emphasized that individual differences in neural stability relate to meaningful differences in autistic traits. Specifically, decreased stability is related to greater parental endorsement of autistic traits measured by the AQ. This individual variation, which is often overlooked in group comparisons, can provide valuable insights into how neural processing contributes to the range of autistic traits and highlights the importance of considering heterogeneity in auditory research. To better capture the heterogeneity among participants, future studies should complement traditional group-level comparisons with analyses that examine associations with autistic traits at the individual level, rather than relying solely on diagnostic group differences.

## 5. Limitations

The present study has limitations that may impact the findings and generalizability of the outcomes. A substantial constraint in the study is the small sample size and restricted diversity among participants. Most participants were Caucasian and had at least one parent with some education beyond high school. Therefore, this sample was not representative of the broader population. Additionally, all participants were required to have a full-scale IQ greater than 80, which limits the applicability of results to individuals with lower IQs. This is particularly important in the generalization of results to the entire autism spectrum, which includes people with a wide range of IQs, including those who are non-verbal or minimally verbal. The relationship between neural stability and autistic traits may differ in individuals with lower cognitive abilities. Additionally, because the model relating SRS-2 total scores with neural stability was insignificant, no further analyses were made to determine if the SRS-2 subscales were differentially related to neural stability.

Importantly, the total number of participants included in each analysis differed. A sensitivity analyses indicated that the AQ model (n = 40) was able to detect effects of approximately β = 0.02–0.53, corresponding to partial r values of approximately 0.24–0.42, reflecting sensitivity to small-to-moderate effects. In contrast, the SRS-2 model (n = 34) required larger effects to detect trait-level associations (β ≈ 0.02; partial r ≈ 0.45) while remaining sensitive to small-to-moderate effects for component and interaction terms (β ≈ 0.02–1.15; partial r ≈ 0.25). Therefore, null SRS-2 findings should be interpreted cautiously, as smaller trait-level effects may not have been detectable while they were detectable in the AQ model.

## 6. Conclusions

This study explored neural stability within the auditory brainstem pathway via ABRs, which shed light on the intricate relationship between stimulus characteristics, response components, and autistic traits. Autistic traits measured by parents’ endorsement on the AQ were significantly related to neural stability. Specifically, less stable neural responses are associated with heightened autistic traits. These findings underscore an important link between the stability of auditory processing of speech and non-speech sounds in shaping autistic traits, hinting at a nuanced relationship with decreased neural stability in response to broader autistic traits, even when exploratory group differences were not significant. A main take away from this study is that it can be beneficial to complement group comparisons with analyses that examine associations with autistic traits at the individual level, especially when investigating highly heterogenous disorders such as autism. Meaningful individual differences can be captured in research by conducting analyses that examine relationships rather than group differences.

Finally, it is crucial to acknowledge the multidimensional nature of autism, characterized by a wide array of traits. Narrowly focusing auditory research on the language deficits associated with autism may inadvertently neglect other facets of autism, including the advantageous ones. Moreover, it is essential to recognize that some features of clinical disorders, such as autistic traits, can manifest within a subclinical population. By quantifying these traits or characteristics across an entire participant cohort, researchers can gain deeper insights into underlying neural mechanisms.

## Figures and Tables

**Table 1:** Participant demographics.

Biological Sex		N	
	Male	21	
	Female	23	
**Education Level (Grade)**			
	Kindergarten	2	
	1st Grade	4	
	2nd Grade	9	
	3rd Grade	2	
	4th Grade	6	
	5th Grade	3	
	6th Grade	4	
	7th Grade	3	
	8th Grade	2	
	9th Grade	2	
	Unknown	7	
**Race**			
White, not Hispanic or Latino		33	
White and Black, not Hispanic or Latino		1	
Native American and White, not Hispanic or Latino		1	
Unknown		9	
**Parental Education**		Mother	Father
High School		2	7
College		2	3
Associate’s		3	2
Bachelor’s		7	6
Graduate School		3	5
Master’s		7	2
Doctorate		8	7
Unknown		12	12

Note. This table provides the demographic information for all participants enrolled in the study, including biological sex, participant education level, race, and parental education level.

**Table 2:** Summary statistics.

	N	Min	Max	Mean	Std. Error	SD
**Participant Variables**						
SRS-2	37	38.00	90.00	59.11	2.55	15.52
AQ	43	0.00	46.00	21.56	1.91	12.53
FSIQ	41	83.00	143.00	110.41	2.02	12.95
PRI	41	88.00	160.00	109.51	2.19	13.99
VCI	41	73.00	134.00	109.24	2.24	14.35
**Neural Stability—Pearson’s R**						
Full Speech	34	0.60	0.98	0.85	0.02	0.09
Onset	34	0.18	0.99	0.88	0.03	0.16
FFR	34	0.62	0.98	0.87	0.02	0.10
Offset	34	0.33	0.98	0.83	0.02	0.14
Click	41	0.59	0.99	0.93	0.01	0.08
**Prestimulus Noise**						
Speech RMS	34	0.02	0.23	0.07	0.01	0.04
Click RMS	41	0.01	0.33	0.09	0.01	0.05

Note. This table provides the n size for each variable measured, as well as the following descriptive statistics: minimum, maximum, mean, standard error of the mean, and standard deviation. SRS-2 = social responsiveness scale-2 total score; AQ = Autism Spectrum Quotient total score; FSIQ = full-scale IQ; PRI = Perceptual Reasoning Index; VCI = verbal comprehension index; Full Speech = neural stability of the entire sABR; Click = neural stability of entire click ABR; Speech RMS = prestimulus noise of the sABR; Click RMS = prestimulus noise of click ABR.

**Table 3:** Bayesian Information Criterion model comparisons.

		BIC	∆BIC
AQ Models			
	Model 1	434.31	0 *
	Model 3	435.32	1.00
	Model 0	436.49	2.18
	Model 2	448.96	14.65
SRS-2 Models			
	Model 0	401.25	0 *
	Model 1	403.15	1.90
	Model 3	408.25	7.00
	Model 2	414.33	13.07

Note. This table reports BIC values and ΔBIC (relative to the best-fitting model). * Indicates the selected best-fitting model.

**Table 4:** Fixed effects from linear mixed-effects model predicting neural stability.

Predictor	Estimate	SE	t	df	*p*	95% CI
Intercept	0.43	0.2	2.2	38	0.035	[0.033, 0.83]
Full sABR	−0.38	0.1	−3.7	135	<0.001	[−0.58, −0.18]
Onset	0.24	0.1	2.4	135	0.019	[0.040, 0.44]
FFR	−0.15	0.1	−1.5	135	0.14	[−0.35, 0.049]
Offset	−0.47	0.1	−4.7	135	<0.001	[−0.67, −0.27]
AQ Total	−0.023	0.0081	−2.8	38	0.0079	[−0.039, −0.0063]

Note. Estimates are from the best-fitting linear mixed-effects—Model 1: neural stability ~ component + trait + (1 | participant). Component effects are coded using sum (effects) coding and are interpreted as deviations from the grand mean across all response components (full sABR, onset, FFR, offset, and click). The click component is included in the model and contributes to the grand mean but is not shown as a separate estimate due to effects coding. Confidence intervals are 95% intervals. Degrees of freedom were estimated using Satterthwaite approximation.

## Data Availability

The data presented in this study are openly available at https://osf.io/r5cax (accessed on 11 May 2026).

## Supplementary Materials

The following supporting information can be downloaded at: https://www.mdpi.com/article/10.3390/brainsci16050535/s1, Supplementary Material S1. Detailed description of neural stability calculation; Supplementary Material S2. Prestimulus Noise; Supplementary Material S3. AQ analysis excluding PT.
