# Supplementary material for: Linking Auditory Brainstem Neural Stability to Parent-Reported Autistic Traits in School-Age Children
Source: Brain Sci. 2026 May 19;16(5):535. doi: 10.3390/brainsci16050535 (PMC13205042; doi:10.3390/brainsci16050535)
Supplement: Supplementary file 1 [file brainsci-16-00535-s001.zip › Supplementary Material S1. Nerual Stability Calculation.pdf]

Supplemental Material S1.

Detailed description of neural stability calculation

Neural stability was quantified using the Pearson product–moment correlation method, which has been used in auditory neuroscience to assess the stability of the ABR over repeated stimulus presentations (also referred to as response consistency; e.g., Anderson et al., 2012; Skoe et al., 2013; Hornickel & Kraus, 2013; Skoe et al., 2015). This approach provides a measure of how well two averaged ABR waveforms match in morphology within the same response time window.

A higher correlation values (r =1) indicate highly repeatable (i.e., low-variability and high stability) neural activity while lower correlation values (r = 0) indicate greater variability (i.e., low stability) in the timing and magnitude of the neural response.

Responses were recorded using alternating-polarity stimulation and dual-channels (right/left). Before calculating the stability of the response, responses were downloaded in the raw data file which saved responses for each channel, into two buffers: one for response evoked by condensation polarity and the other for rarefaction polarity. Because of this, we correlated a subset of trials to ensure that the resulting stability metric reflects true neural stability rather than artifact from stimulus polarity differences, channel noise, or order of data collection. Below a detailed description of the steps taken and a visual representation is provided to explain how neural stability was calculated in the current study.

Speech-evoked ABRs and click ABRs were collected in two channels (right and left), each consisting of 3,000 sweeps, combined from two, 1500 sweep averages collected over two runs (1st and 2nd repetitions). For the sABR, binaural stimuli alternated between condensation and rarefaction polarities and the resulting responses were stored in separate buffers (buffers 1 and 2). When responses from the two 1500 sweep runs are averaged within each buffer, polarity-specific timing differences in neural firing are observed between the two buffers. As shown in Figure S1 A, waveforms averaged separately by stimulus polarity have shifted peak latencies and amplitudes. Subsequently, if responses evoked by a single polarity are averaged and then correlated with each other, the correlation coefficient could be reduced (i.e.,less stable) due to polarity-dependent differences rather than true neural variability.

To avoid morphological differences arising due to stimulus polarity rather than differences in neural responses, we averaged responses from right channel condensation buffer (buffer 1; 1500 sweeps) with the left channel, rarefaction buffer (buffer 2; 1500 sweeps). Said another way, each channel contained two buffers of 1500 sweeps (buffer 1: condensation evoked sweeps and buffer 2 : rarefaction evoked sweeps) and to remove polarity-specific timing shifts, we averaged condensation from one channel with rarefaction from the opposite channel, and vice versa. As shown in Figure 1B averaging across polarity produces two overlapping responses. This produced two subaverage waveforms, each containing equal numbers (3000) of independent (nonoverlapping) sweeps, that are matched in stimulus polarity composition and recording order.

Although click ABRs were collected only in condensation polarity, the same averaging procedure (using right/left channels and 1st /2nd buffers) was applied for methodological consistency and to eliminate channel- or recording order differences.

Figure S1

Averaging Methods

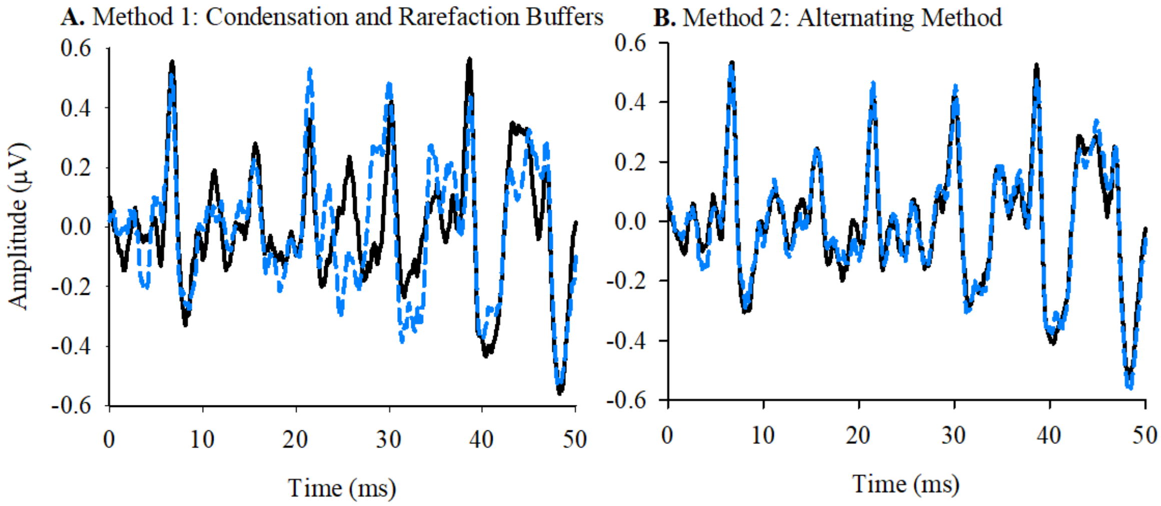

Note. Figure S1 depicts data recorded from one participant but averaged together in two different methods. Panel **A** displays two waveforms: an average condensation waveform (3000 sweeps) evoked using condensation polarity (black solid line) and an average rarefaction waveform (3000 sweeps; blue broken line). Panel B displays the alternating waveforms, in which the responses evoked by condensation and rarefaction sweeps are averaged together.

The Pearson product–moment correlation (Pearson's r) was computed between the two subaverage waveforms (3000 sweeps). This is standard metric of *intertrial response consistency*, has been used to quantify the degree to which repeated presentations of the same stimulus evoke reproducible neural activity (Anderson et al., 2012; Hornickel & Kraus, 2013; Skoe et al., 2015). The metric is sensitive to both amplitude and latency jitter between trials, making it a robust index of neural stability.

Figure S2

Steps to Calculating Neural Variability- Step 1

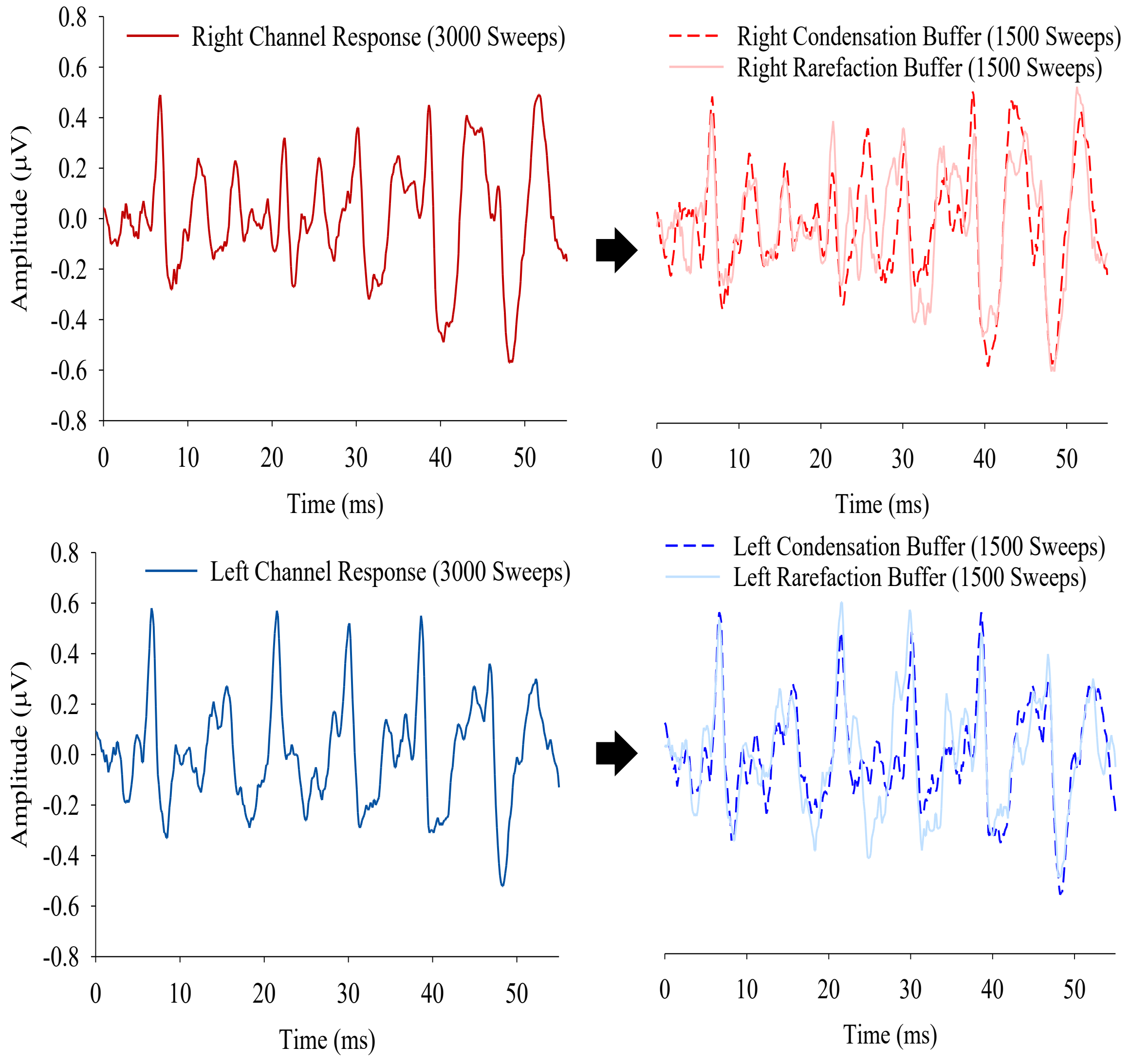

Note. Figure S2 displays the first step involved in calculating neural variability, which was to segregate the average waveforms from the right and left channels from the condensation and rarefaction buffers.

Figure S3

Steps to Calculating Neural Variability- Step 2

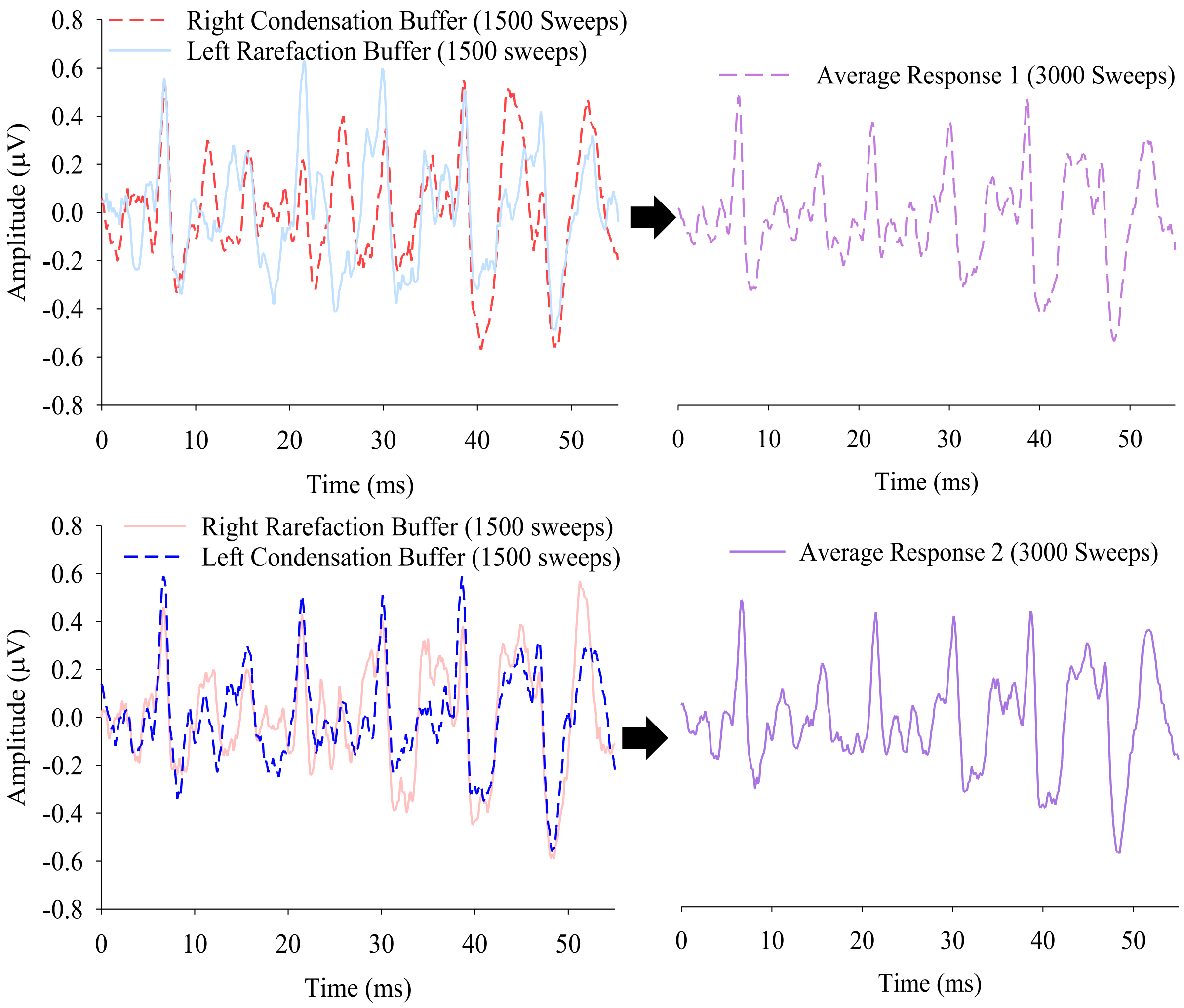

Note. Figure S3 illustrates the second step in calculating neural variability. As shown above, the rarefaction and condensation buffers (each comprising of 1500 sweeps) from opposite channels were averaged to create two distinct waveforms, labeled as Average response 1 and 2. Each average waveform contains 3000 sweeps and integrates responses recorded from right and left channels.

Below are example sABR waveforms that depict a correlation that indicates a stable response (Figure S4A) and a poor correlation, that indicates a less stable response as evident by the poorly matched morphology between the two subaverages (Figure S4B).

Figure S4

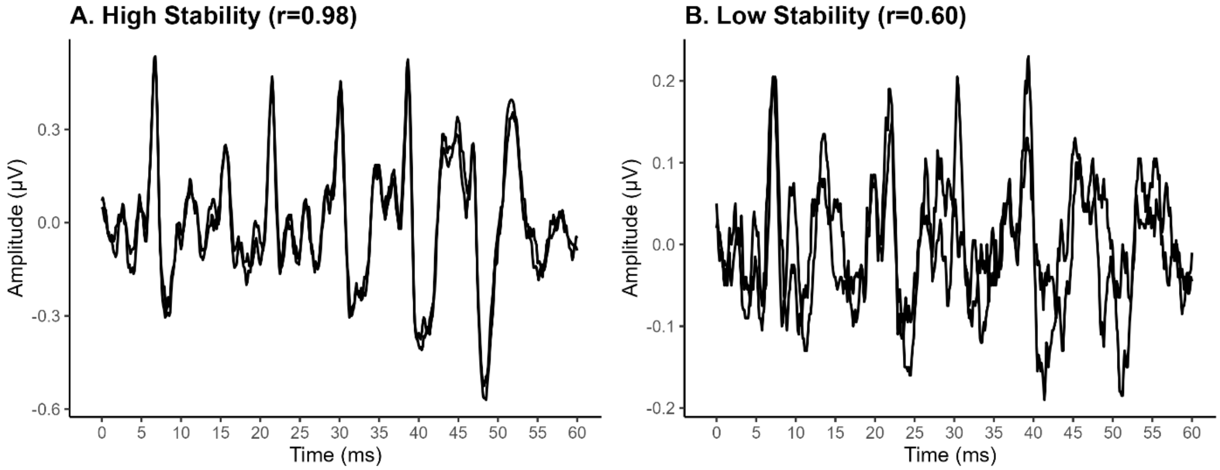

Note. Figure S4 depicts two examples from a participant with a highly stable full sABR response component (panel A) and a participant with a less stable full sABR response component (panel B).
