# Supplementary material for: Linking Auditory Brainstem Neural Stability to Parent-Reported Autistic Traits in School-Age Children
Source: Brain Sci. 2026 May 19;16(5):535. doi: 10.3390/brainsci16050535 (PMC13205042; doi:10.3390/brainsci16050535)
Supplement: Supplementary file 1 [file brainsci-16-00535-s001.zip › Supplementary Material S2. Prestimulus Noise.pdf]

<u>Supplemental Material S2</u>

Prestimulus Noise Correlations.

Table S2.1 The correlations between prestimulus noise and all response components

Component	n	r	p	95% CI
Full sABR	34	-0.01	0.98	[-0.35- 0.34]
Onset	34	0.13	0.48	[-0.23 - 0.45]
FFR	34	0.05	0.80	[-0.30 - 0.38]
Offset	34	-0.12	0.51	[-0.44 - 0.23]
Click	40	-0.03	0.85	[-0.34 - 0.28]

Note. Correlation analyses are reported between click-evoked ABR responses and click prestimulus noise, and between speech-evoked ABR responses and speech prestimulus noise

Table S2.2 The correlations between prestimulus noise and participant variables: IQ, age, AQ and SRS-2

Prestimulus Noise	Participant variable	n	r	p	95% CI
Speech	Age	33	-0.43	0.01	[-0.68 - -0.10]
Speech	VCI	31	0.24	0.19	[-0.12 - 0.55]
Speech	AQ	33	-0.14	0.44	[-0.46 - 0.21]
Speech	SRS	30	-0.22	0.25	[-0.53 - 0.16]
Click	Age	40	-0.48	0.00	[-0.69 - -0.20]
Click	VCI	37	-0.15	0.37	[-0.45 - 0.18]
Click	AQ	40	-0.04	0.81	[-0.35 - 0.27]
Click	SRS_	34	0.02	0.89	[-0.32 - 0.36]

Note. Correlation coefficients are reported between prestimulus noise (measured separately for click-evoked and speech-evoked ABRs) and each participant-level variable.
