# Supplementary material for: Linking Auditory Brainstem Neural Stability to Parent-Reported Autistic Traits in School-Age Children
Source: Brain Sci. 2026 May 19;16(5):535. doi: 10.3390/brainsci16050535 (PMC13205042; doi:10.3390/brainsci16050535)
Supplement: Supplementary file 1 [file brainsci-16-00535-s001.zip › Supplementary Material S3. AQ analysis excluding PT.pdf]

Supplemental Material S3

Analyses Excluding Participants who completed Adult AQ version

To confirm that the participants (n=4) who completed the Adult AQ did not skew the results of the model, the analysis was repeated with the data from these participants excluded. The AQ results were unchanged. AQ remained a significant negative predictor of neural responses ($\beta = -0.021$, SE = 0.008, $t = -2.5$, $p = 0.017$). This indicates that the observed association between a greater endorsement of AQ traits and reduced neural stability is not driven by a small subset of participants who completed the survey on their own as opposed to their parents.

Table S3. Linear Mix Model Results Comparison

Term	Estimate	Std. Error	Statistic	df	p.value
Excluding Self-Reporting Participants					
(Intercept)	0.401	0.210	1.911	32.321	0.065
Component1	-0.361	0.109	-3.320	114.554	0.001
Component2	0.228	0.109	2.092	114.554	0.039
Component3	-0.150	0.109	-1.382	114.554	0.170
Component4	-0.503	0.109	-4.624	114.554	0.000
AQ_Total	-0.021	0.008	-2.508	32.623	0.017
Including All Participants					
(Intercept)	0.432	0.197	2.193	37.598	0.035
Component1	-0.379	0.101	-3.745	134.881	0.000
Component2	0.240	0.101	2.374	134.881	0.019
Component3	-0.151	0.101	-1.494	134.881	0.137
Component4	-0.471	0.101	-4.659	134.881	0.000
AQ_Total	-0.023	0.008	-2.805	38.258	0.008

**note. Table includes results from the model that excluded participants who completed the Adult-AQ, vs the model that is reported in the main text, which includes all participants. The statistical model compares each component relative to the overall average. Only four of the five components are shown in the table. The fifth component is not missing; it is mathematically determined by the other components. The intercept represents the average value across all five components.*
